# The Paraguay Tea Tree

**Published:** 1884-08

**Authors:** 


### THE PARAGUAY TEA TREE.

THE superintendent of gardens and grounds attached to the United States Department of Agriculture mentions in his last report that the department has recently had numerous inquiries regarding the feasibility of growing in the United States a plant similar to the *Ilex paraguayensis*, or Paraguay tea tree, from which the leaves are stripped and used in infusion as an article of food under the name of *mate*, and gives the following description of the cultivation of the tree, and the method employed in the preparation of this article. In rich soils the tree will attain a height of from seventy to ninety feet ; it is said to be confined to mountain slopes, never appearing on table lands nor the broad plains which skirt the river beds, while it is plentiful in all the moist valleys that divide the waters of the Parana and Paraguay rivers. For the preparation of *mate* proper, the leaves are dried, or roasted in cast-iron pans set in brickwork and heated by fires underneath ; when the leaves are sufficiently heated, they are pounded in stamping mills worked by water or steam power until reduced to powder, and then packed in bags by means of presses. There are three qualities or sorts of yerba known in the South American markets. The best is said to be prepared from the young leaves when they are about half expanded from the bud, called *caa-cuys;* the second consists of the full-grown leaves, carefully picked and separated from the twigs, and frequently the midrib and veins of the leaves are removed ; this is called *caa-mira;* the third is the *caa-gnaza* or *Yerva de Palos*, made from older leaves, carelessly broken up with the small branches and leaf-stalks, all of which undergo the roasting and pounding process together. The leaves are also collected and dried in a similar manner to that adopted in the preparation of Chinese tea. This is called *mate* in leaf, and is prepared for use by infusion, and taken with milk and sugar in the same way as ordinary tea. *Mate* in powder is also prepared by infusion, by putting into a small vessel about an ounce of the powder and pouring boiling water over it. As the fine dust does not fall to the bottom but remains suspended in the water, the *mate* is taken by means of a sucker, that is, a tube terminating in a small hollow ball pierced with very fine holes. *Mate* contains theine, the same active principle as tea and coffee, but is not possessed of their volatile and empyreumatic oils ; it contains less essential oil, more resin than coffee, but less than is found in tea. Chemical analyses show that it contains nearly double the quantity of theine that the same weight of grains of coffee, and about the same quantity as tea leaves. The Brazilians recommend *mate* as a nourishing, warm, aromatic, stimulating, and very cheap beverage, its extreme cheapness being a guarantee of its genuineness, as it is not worth adulterating.

